# Histopathological Insights Into Asymptomatic Gallstone Disease: Justifying Prophylactic Cholecystectomy in a High-Risk North Indian Population

**DOI:** 10.7759/cureus.101925

**Published:** 2026-01-20

**Authors:** Tamish Sharma, Mathew Vadukoot Lazar, Rajesh Kumar Sahu, Praveen Jose, Avneesh Kumar, Sadaf Khan, Eishu Verma, Preethi Jose

**Affiliations:** 1 General Surgery, Shri Guru Ram Rai Institute of Medical and Health Sciences (SGRRIMHS), Dehradun, IND; 2 Gastroenterology and Hepatology, Lifecare Hospital, Mussafah, Abu Dhabi, ARE; 3 Pathology, Shri Guru Ram Rai Institute of Medical and Health Sciences (SGRRIMHS), Dehradun, IND; 4 Radiodiagnosis, George Elliot Hospital, Nuneaton, GBR

**Keywords:** asymptomatic gallstones, cholecystectomy, cholelithiasis, chronic inflammation, gallbladder cancer, histopathology, laparoscopic intervention, metaplasia, north india, prophylactic surgery

## Abstract

Introduction

While often considered benign, asymptomatic gallstone disease in high-incidence regions such as North India may carry a risk of progression to gallbladder carcinoma, warranting further study. This prospective cross-sectional descriptive observational study aimed to describe the histopathological features of incidentally diagnosed asymptomatic gallstones in a high-risk North Indian population and explore whether these findings support selective prophylactic cholecystectomy to prevent malignant progression

Methods

A prospective, cross-sectional, descriptive, observational study was conducted on 99 patients undergoing elective laparoscopic cholecystectomy at a tertiary care center. Asymptomatic status was strictly defined as the absence of biliary pain or symptoms directly linked to gallstones. Patients with non-specific dyspepsia were included only if they had not experienced biliary colic symptopms. Detailed intraoperative findings and postoperative outcomes were documented. Histopathological analyses were performed to identify inflammatory, metaplastic, and neoplastic changes.

Results

Despite being asymptomatic, 36 patients (36.3%) had chronic inflammatory changes, and 32 patients (32.2%) showed pyloric metaplasia. High-grade dysplasia or malignancy was detected in 3 (3%) patients. Age was a significant predictor of pathological severity (p = 0.014). Laparoscopic surgery was safe, with minimal complications, and enabled early detection of pre-malignant conditions.

Conclusion

Our findings suggest that elective laparoscopic cholecystectomy may be considered in asymptomatic patients from high-risk regions. Histological findings reveal that silent gallstones are not always clinically insignificant, especially in the Indian subcontinent, where gallbladder cancer is endemic.

## Introduction

Cholelithiasis, or gallstone disease, is one of the most common disorders of the biliary tract, affecting an estimated 10-20% of the adult population in Western nations [1]. The incidence is increasing globally and is no longer restricted to high-income countries. In India, especially in northern regions, the burden of gallstone disease has risen significantly in recent decades, primarily due to dietary changes, urbanization, and improved access to diagnostic imaging, particularly ultrasonography [2].

Gallstones are broadly classified into two categories: symptomatic and asymptomatic. Approximately 60-80% of individuals with gallstones remain asymptomatic throughout life [3,4]. Asymptomatic gallstones are characterized by the presence of gallstones without biliary pain or related symptoms. These "silent gallstones" are typically diagnosed incidentally during evaluation for unrelated abdominal complaints, gynecological assessments, or routine health check-ups. While many such cases do not require immediate intervention, some may progress to develop serious complications such as acute cholecystitis, choledocholithiasis, gallstone pancreatitis, or, more importantly, gallbladder carcinoma.

The association between gallstone disease and gallbladder cancer is particularly strong in the Indian subcontinent, which contributes nearly 10% of the global disease burden, with the highest incidence in northern and northwestern India. This oncogenic transformation is thought to be caused by multiple risk factors, including chronic inflammation, recurrent mechanical damage from gallstones,* Salmonella *Typhi carrier status, and exposure to environmental heavy metals. It has been proposed that chronic cholelithiasis can be a preexisting lesion, and the latent phase is 10 to 15 years before dysplasia advances to invasive carcinoma [5].

This study aims to generate histopathological evidence from a high-risk population in North India, characterized by a gallbladder cancer incidence exceeding 5 per 100,000 and influenced by dietary factors, heavy metal exposure, and Salmonella carriage, to evaluate whether asymptomatic gallstones exhibit premalignant changes that could support selective prophylactic cholecystectomy. High-risk groups include those with large stones (>3cm), wall thickening, or anomalies like pancreato-biliary maljunction, even without symptoms. These findings can be applied by clinicians in similar endemic areas through risk-stratified screening and multicenter collaboration for larger cohorts

Given the oncological consequences, interest in discussing prophylactic surgery options is increasing. Laparoscopic cholecystectomy has emerged as the gold standard for gallbladder removal, owing to its minimally invasive nature, favorable safety profile, and cost-effectiveness [6].

Some surgeons in high-risk areas like North India and Chile advocate for early elective cholecystectomy even in asymptomatic patients, especially those with some high-risk imaging features, such as large stones (>3 cm), gallbladder wall thickening, or anatomic anomalies like pancreaticobiliary maljunction and positive family history. These features may be present in the absence of typical biliary symptoms, yet may warrant consideration of prophylactic surgery because of an elevated risk of malignancy. Nevertheless, this approach remains controversial due to concerns about overtreatment, surgical complications, and the lack of definitive evidence in international guidelines. The 2007 Cochrane review on the topic concluded that the evidence to either recommend or not recommend prophylactic surgery in asymptomatic persons is insufficient. [7].

Although no global consensus exists, the epidemiological peculiarity of gallstone-associated malignancy in India necessitates reexamining current clinical practices. Early diagnosis and surgery could provide a critical window to prevent malignant progression in asymptomatic patients. Moreover, due to advancements in laparoscopic surgery, the risk of complications has decreased considerably, and prophylactic surgery may be an option in carefully selected cases [8].

Review of literature

Gallstone disease is the most common biliary tract disorder and is strongly associated with gallbladder carcinoma, particularly in high-burden regions such as northern India. Although they are usually asymptomatic, gallstones have been suggested as a significant risk factor of gallbladder cancer, with particular focus on populations with a long history of chronic cholelithiasis (often asymptomatic for extended periods) [5]. Epidemiological studies have established an inverse correlation between higher rates of cholecystectomy and lower mortality due to gall bladder carcinoma. Indicatively, mortality due to gallbladder cancer has decreased in places like Scotland, England, Wales, Canada, and the United States, where elective cholecystectomy is more commonly done. However, Sweden has recorded a 33 percent% increase in mortality due to gallbladder cancer in the past ten years. It has been estimated that one death from gallbladder cancer could be prevented for every 100 cholecystectomies performed, highlighting the potential role of surgical intervention in disease prevention [9].

The decision to offer cholecystectomy in asymptomatic patients is complex. Although some studies caution against the excessive use of cholecystectomy due to potential surgical complications and altered bile physiology, others suggest that cholecystectomy may influence the risk of gastrointestinal malignancies, including gastric and colorectal cancers, with effects varying by population and geographic region [9-11]. A large retrospective analysis comparing patients with gallstone disease who underwent cholecystectomy to population-based controls demonstrated an increased prevalence of gastric carcinoma in the cholecystectomy group, suggesting a possible association mediated by bile reflux and chronic gastric mucosal injury. Multivariate regression analysis revealed lower survival in the gallstone and cholecystectomy group, with a hazard ratio of 6.66 (p = 0.003), suggesting that gastric mucosal damage and bile reflux interact in a complex manner [10]. On the other hand, a systematic review and meta-analysis study by Yu et al. proved a lower risk of colorectal cancer among people with gallbladder disease who have undergone cholecystectomy, especially the Asian population, with a relative risk of 0.66 (p < 0.001) [11]. These results highlight the geographical and ethnic diversity in post-cholecystectomy outcomes and the need for clinical recommendations at the regional level.

Nearly two-thirds of gallstone cases diagnosed in India are asymptomatic, lacking biliary symptoms such as colic or cholecystitis. Despite the absence of symptoms, longitudinal studies have shown that 15-25% of such patients may develop complications over a 10-year period, including pancreatitis, empyema, perforation, and malignant transformation. Although the operative mortality of laparoscopic cholecystectomy done electively (without emergency) is low (less than 0.5%), it may be pretty high in older patients or in cases complicated by conditions necessitating emergency intervention. Conservative management of asymptomatic cholelithiasis has been recommended in traditional decision-analytic studies. Nevertheless, today there are specific clinical situations, including porcelain gallbladder, suspected malignancy, or non-functioning gallbladder, that provide clear indications for early surgery, independent of the presence or absence of symptoms [12,13].

Efforts have increasingly focused on identifying subgroups that could benefit from prophylactic cholecystectomy, such as younger people with increased cumulative cancer risk, thickening of the gallbladder wall, large stones (>3 cm), or anatomic anomalies, such as pancreaticobiliary maljunction. In high-incidence areas, specifically in northern India, the benefits of surgery may outweigh the associated risks [3]. Early onset of gallstones is observed in the case of Indian women, and research estimates that 60-90% of gallbladder cancer cases in India have a history of a preceding gallstone. Long-standing or large stones are linked to increased rates of complications, such as xanthogranulomatous cholecystitis and Mirizzi syndrome, which are associated with an increased risk of malignancy [14].

In India, gallbladder cancer carries an exceptionally high disease burden, especially among women in the northern and northeastern regions, where it is the most common gastrointestinal malignancy. In India, the interval from symptom onset to diagnosis is about 34 months, substantially longer than the 10 months reported in the UK, contributing to delayed diagnosis and poor survival. The statistics on the incidence of gallbladder cancer as the primary cause of malignant obstructive jaundice in the city of Lucknow and the adjacent regions demonstrate that early detection methods are essential [15].

Environmental exposures have been implicated in gallbladder carcinogenesis in India. In an Indian-Japanese study, higher levels of heavy metals, such as lead and chromium, were observed in the gallbladder tissues of Indian patients compared with Japanese patients. These results indicate that environmental exposure may have a co-carcinogenic effect, which may be exacerbated by diet and microbial factors, including chronic *Salmonella *Typhi carriage [5]. As an example, the incidence in Kamrup, Assam, is 14 cases per 100,000 women yearly, versus 7 per 100,000 in Chile, where prophylactic cholecystectomy has already become part of the official health practice [16].

Overall, in India, the high usage and accessibility of abdominal ultrasonography has resulted in a higher prevalence of asymptomatic gallstones detected by accident. Early diagnosis offers a unique opportunity to consider elective surgical intervention, especially in endemic areas, where the development of severe pathology often accompanies the natural history of gallstones. Therefore, although a global agreement has not yet been reached, regional epidemiological data can serve as a strong argument for reconsidering the importance of prophylactic cholecystectomy in the Indian context, particularly in asymptomatic patients whose risks can be predicted [2].

Research objectives

This prospective cross -sectional descriptive observational study aimed to describe the clinical, surgical, and histopathological spectrum of asymptomatic gallstone disease in patients who underwent cholecystectomy at a tertiary care unit in North India. In particular, the primary objective was to describe histopathological alterations in the gall bladder specimens of these patients, including chronic inflammation, metaplasia, or malignancy, and explore their association with demographic factors to assess the potential justification for prophylactic cholecystectomy in a high-risk population.

## Materials and methods

This was a prospective, cross-sectional, descriptive, observational study conducted over 18 months in the Department of General Surgery at Shri Mahant Indiresh Hospital, a tertiary care center in Dehradun, Uttarakhand, India. The aim was to determine the clinical, surgical, and histopathological results of laparoscopic cholecystectomy in patients diagnosed with non-symptomatic gallstone disease.

The sample size was calculated using the below-mentioned formula to estimate the proportion of rare histopathological changes (e.g., malignancy, expected p=0.01)with a 95%confidence interval and ± 2% margin error, aligning with the study's focus on detecting pre-malignant/neoplastic alterations in a prospective observational design. The expected malignancy rate (p=0.01) was based on incidental gallbladder carcinoma rates reported in Indian North Indian cohorts, such as 1.9%.

$n = \frac{Z_{\alpha/2}^{2} \times p (1 - p)}{E^{2}}$

where n is the required sample size for the study, Z_α/2 _is the Z-score (critical value) corresponding to the desired confidence level, pis the expected prevalence, 1−p is the complement of the population proportion, and E is the desired margin of error. 

All patients referred to the surgical outpatient department who were incidentally diagnosed with asymptomatic cholelithiasis were eligible to participate in the study. Asymptomatic status was defined as the absence of biliary pain or any individual symptom linked to gallstones. Patients who presented with dyspepsia without biliary colic were included only if no biliary symptoms were present. Individuals who had been diagnosed with gallbladder malignancy previously, with symptomatic disease, extreme age, or during pregnancy were not included in the study.

A consecutive sampling design was used, enrolling all eligible patients in order of presentation until the target sample was met. This non-probability approach was chosen for its feasibility in a clinical setting and for minimizing selection bias while ensuring ethical inclusion of all qualifying cases. Variables collected included demographics (age, gender, comorbidities via patient history and records), ultrasound indications (from referral notes), intraoperative findings (adhesions, calculi type via surgical logs), postoperative outcomes (complications, hospital stay via ward records), histopathological features (inflammation, metaplasia, neoplasia via standardized lab reports), and the presence of any high-risk pathological changes. Data reliability was ensured through standardized proformas, double-entry verification, and histopathological review by two pathologists to assess inter-observer agreement. Validity was maintained by adhering to institutional ethics and excluding symptomatic cases

The research was conducted in accordance with institutional ethics and the ethics committee's guidelines. Every participant signed a written informed consent form after being informed of the study's purpose and nature. All patients underwent laparoscopic cholecystectomy, which was the preferred surgical modality. They were also informed and gave consent to the possibility of conversion to open cholecystectomy, should it be required. Only surgeons at the assistant professor level or higher performed all procedures to ensure surgical consistency and reduce variability.

The intraoperative observations were documented in detail on a patient-by-patient basis, including the type of technical difficulty encountered (e.g., dense adhesions, anatomical variations encountered during Calot triangle dissection). Every gallbladder specimen excised underwent routine histopathological analysis to assess epithelial alterations, inflammatory conditions, metaplasia, and the presence of pre-cancerous or cancerous changes. Statistical analysis included Chi-square tests (with degrees of freedom and Cramér's V where applicable) and logistic regression to identify predictors of severe histopathological changes.

## Results

The study comprised 99 participants with a diagnosis of asymptomatic gallstone disease. The study population had a mean age of 45.76 years (range: 15-81 years), with 70 females (70.7%) and 29 men (29.3%). The 46-60-year age group had the highest incidence of gallstones, 34 patients (34.3%), followed by the 31-45-year age group, 30 patients (30.5%). Age groups were categorized into 15-year intervals to align with standard epidemiological stratification for gallstone disease progression and cancer risk across stages (Table 1).

**Table 1:** Age-group and gender distribution of the study participants (n=99)

	Female		Male		Total	
Age Group	Frequency	%	Frequency	%	Frequency	%
15-30	16	22.8	2	6.9	18	18.1
31-45	19	27.1	11	38	30	30.5
46-60	24	34.2	10	34.5	34	34.3
≥ 61	11	15.7	6	20.6	17	17.1
Total	70		29		99	

In both genders, over half of patients (20 (69%) males and 40 (57.2%) females) had no comorbidities. Among individuals with comorbid diseases, the most prevalent conditions were hypertension (14 (20%) females and 4 (13.7%) males), diabetes mellitus (8 (11.4%) females and 2 (6.9%) males), and hypothyroidism (5 (7.2%) females and 2 (6.9%) males) (Table 2).

**Table 2:** Comorbidities among the study participants (n=99) * = includes hypothyroidism, bronchial asthma, coronary artery disease, chronic kidney disease and other less frequent comorbidities

	Female		Male	
Comorbidities	Frequency	%	Frequency	%
Hypertension	14	20	4	13.7
Diabetes Mellitus	8	11.4	2	6.9
Hypothyroidism	5	7.2	2	6.9
None	40	57.2	20	69
Others*	3	4.2	1	3.5
Total	70		29	

Dyspepsia was the most frequent reason for ultrasonography in 37 (37.3%) patients, and it was particularly common in 20 (68.9%) males. The most common reasons for abdominal sonography in 22 (31.4%) females were gynecological symptoms or diagnoses (menstrual irregularities, pelvic pain, or fibroids), general examinations (routine health screenings) in 18 (25.7%) females, and dyspepsia in 17 (24.2%) females (Table 3).

**Table 3:** Reasons/ indications of undergoing ultrasound (n=99)

	Female		Male		Total	
Indication for ultrasound	Frequency	%	Frequency	%	Frequency	%
General checkup	18	25.7	3	10.3	21	21.2
Dyspepsia	17	24.2	20	68.9	37	37.3
Gynecological problem	22	31.4	0	0	22	22.2
Nausea	6	8.5	6	20.6	12	12.1
Urinary problem	7	10	0	0	7	7
Total	70		29		99	

The majority of patients (72 (72.7%)) were discharged between 4 and 6 days, while hospital stays ranged in length. In particular, 26 patients (26.3%) remained hospitalized for 4 days, 27 (27.3%) remained hospitalized for 5 days, and 19 (19.2%) patients stayed for 6 days. As shown in Table 4, two patients (2%) required hospitalization for more than 9 days, whereas only 3 patients (3%) were discharged after 2 days.

**Table 4:** Length of hospital stay (n=99)

	Female		Male		Total	
Length of hospital stay (days)	Frequency	%	Frequency	%	Frequency	%
2	2	2.8	1	3.4	3	3
3	10	14.2	3	10.3	13	13.2
4	14	20	12	41.3	26	26.3
5	20	28.5	7	24.1	27	27.3
6	16	22.8	3	10.3	19	19.2
7	8	11.4	1	3.4	9	9
9	0	0	1	3.4	1	1
12	0	0	1	3.4	1	1
Total	70		29		99	

Patients were followed up to evaluate long-term outcomes, including post-cholecystectomy syndrome; however, the primary outcome of interest was histological. Thirty-two (32.3%) patients were lost to follow-up after the initial postoperative visit, whereas 67 (67.7%) patients completed follow-up at 3 months after surgery (Table 5).

**Table 5:** Post-surgery follow-up of the study participants(n=99).

	Female		Male		Total	
Follow-up Duration (Months)	Frequency	%	Frequency	%	Frequency	%
1	23	32.8	9	31	32	32.3
3	47	67.2	20	69	67	67.7
Total	70		29		99	

Laparoscopic cholecystectomy was performed on each patient. During surgery, numerous calculi were present in 65(65.6%) patients, and 94 (94%) patient had enlarged gallbladders. In 34 (34.4%) cases, a single big calculus was discovered. In 40(40%) cases, dense adhesions involved the anterior abdominal wall, duodenum, liver, or omentum. Thirty (30.3%) of cases had adhesions near Calot's triangle, making dissection more difficult. Frozen Calot’s anatomy was observed in 10% of cases, with one instance of intraoperative biliary leakage.

**Figure 1:**
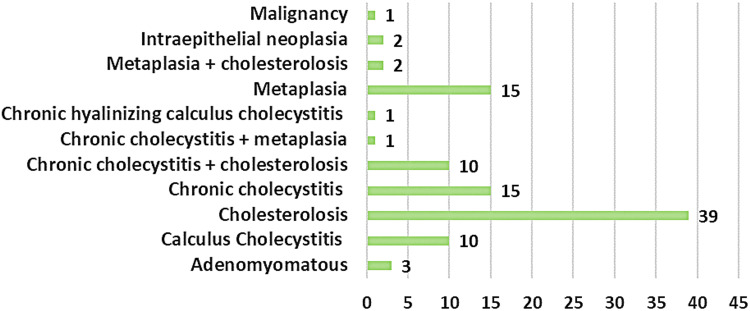
Histopathology examination findings of the cholecystectomy specimen of the study participants (n=99)

Cholesterolosis was the most frequent histological finding, observed in 39 patients (39.4%), followed by chronic cholecystitis in 15 patients (15.2%), and combined cholesterolosis with chronic cholecystitis in 10 patients (10.1%). Adenomayomatosis hyperplasia was seen in 3 patients (3.0%), biliary intraepithelial neoplasia in 2 patients (2.0%), metaplasia in 15 patients (15.2%), and one case (1.01%) of T2 gall bladder adenocarcinoma (Figure 1).

A significant proportion of gallbladders showed histopathological epithelial alterations. The most frequent epithelial changes observed were partial or total ulceration in 32 patients (32.3%), hyperplasia in 19 patients (19.3%), and focal erosion in 22 patients (22.3%); the remaining cases demonstrated no remarkable epithelial alterations (Table 6).

**Table 6:** Epithelial changes in the gall bladder of the cholecystectomy specimen of the study participants (n=99)

Epithelial Changes	Frequency	%
Denuded mucosa	5	5.05
Focal erosion	22	22.3
Hyperplasia	19	19.3
Mucosa into folds	12	12.1
Mucosa into folds, high-grade dysplasia	2	2.02
Partial ulceration	19	19.1
Ulceration	13	13.2
Normal	6	6.06
Partly atrophic and hyperplastic	1	1.01
Total	99	

Notably, there were inflammatory infiltrates in the lamina propria, with 36 (36.3%) of patients exhibiting chronic inflammation. Acute-on-chronic inflammation was observed in 16 patients (16.1%), moderate chronic infiltrates in 16 (16.1%), and mild chronic infiltrates in 18 (18.1%), while 12 patients (12.1%) showed no significant inflammation (Table 7).

**Table 7:** Condition of Lamina Propria among the Cholecystectomy specimens of the study participants(n=99).

Lamina Propria	Frequency	%
Acute and Chronic inflammatory infiltrate	16	16.1
Chronic inflammatory	13	13.1
Chronic inflammatory infiltrate	36	36.3
Mild Chronic inflammatory infiltrate	18	18.1
Moderate Chronic Inflammation	16	16.1
Total	99	

In a considerable number of cases, subepithelial gland alterations were noted. Histological analysis demonstrated pyloric metaplasia in 32 specimens (32.2%), while 12 (12.1%) were unremarkable and 43 (43.4%) appeared normal (Table 8).

**Table 8:** Changes in the subepithelial glands of the cholecystectomy specimen of the study participants (n=99)

Subepithelial Glands	Frequency	%
Extensive polypoidal pyloric metaplasia	8	8.08
Focal pyloric Metaplasia	4	4.04
Pyloric metaplasia	32	32.2
Unremarkable	12	12.1
Normal	43	43.4
Total	99	

Rokitansky-Aschoff (RA) sinuses were present in 55 specimens (55.5%), while 20 (20.2%) demonstrated normal sinuses and 16 (16.1%) showed no discernible RA sinuses; complete sinus absence was observed in 8 cases (8.0%) (Table 9).

**Table 9:** Status of Rokitansky-Aschoff sinuses among the cholecystectomy specimens of the study participants (n=99)

Rokitansky-Aschoff sinuses	Frequency	%
Absent	8	8.08
Normal	20	20.2
Not identified	16	16.1
Present	55	55.5
Total	99	

Examination of the muscularis propria revealed thickening with inflammation in 16 cases (16.1%), variable thickening in 28 cases (28.3%), and chronic inflammation in 23 cases (23.2%); only 12 specimens (12.1%) were histologically normal (Table 10).

**Table 10:** Pathological findings of muscularis propria (n=99)

Muscularis propria pathology	Frequency	%
Chronic inflammatory	23	23.2
Thickened with chronic inflammatory	20	20.2
Variably thickened	28	28.3
Variably thickened with chronic inflammatory	16	16.1
Normal	12	12.1
Total	99	

In all cases, pathological alterations in the serosa were noted. Fibrosis with vascular congestion was present in 43.4% of cases, while 20.2% showed fibrosis alone and 36.3% demonstrated isolated vascular congestion (Table 11).

**Table 11:** Pathological findings of the serosa (n=99)

Serosa	Frequency	%
Congested dilated Blood Vessels	36	36.3
Fibrosis	20	20.2
Fibrosis Congested with dilated Blood Vessels	43	43.4
Total	99	

Laparoscopic cholecystectomy was performed in all cases, with no conversion to open surgery. Postoperative complications were minimal, with one case of a retained drain and one port-site infection, both of which resolved without sequelae. Ultrasound indications were significantly associated with the final histological diagnosis (X^2 ^(df=12, N=99)=23.64, P=0.033, Cramer's V=0.35). However, neither comorbidity status (X^2 ^(6, N=99) =10.89, P=0.117, Cramer's V=0.23) nor gender (X^2 ^(df=3, N=99) =1.39, P=0.707, Cramer's V=0.12) was statistically associated with histological findings.

Key quantitative variables included age (mean ± SD: 45.76 ± 16.4 years), length of hospital stay (median(IQR): 5(4-6) days), and follow-up duration (median(IQR): 3(1-3) months). An exploratory binary logistic regression model was conducted to assess potential predictors of severe histopathological alterations(defined as the presence of metaplasia or neoplasia/malignancy as the binary outcome). The model included age (continuous), gender(binary), length of hospital stay (continuous), follow-up duration (continuous), and ultrasound indication. Age was a significant predictor (p=0.014), while other factors were not (p>0.05). The model's Pseudo-R² (Nagelkerke) was 0.099, indicating low explanatory power. Given the sample size (n=99) and ~35 events, the events per variable ratio (~4.4) was below recommended thresholds, thus limiting reliability and potentially violating assumptions such as adequate power for stable estimates. No model diagram is provided, as this is a standard multiple binary logistic regression without complex structures. 

## Discussion

This study assessed the surgical and histological implications of preventative cholecystectomy in patients with asymptomatic (silent) gallstones in a tertiary care setting in North India. The results validate the danger of ignoring asymptomatic gallstones, especially among high-risk groups [1].

All of the patients had a normal postoperative recovery, with laparoscopic cholecystectomy being the most preferred method of surgery. Postoperative complications were minimal, with one case of a retained drain and one port-site infection, both of which were treated promptly without any long-term consequences. These findings align with the literature that indicates that early elective laparoscopic cholecystectomy is of low morbidity, primarily when performed in areas with a high incidence of gallbladder cancer, such as Chile and North India [3].

The highest prevalence and clear female dominance (70.7%) were observed in the 46-60 age group. Consistent with existing literature, this study confirms the association between increased cholesterol saturation, estrogen-related hormonal effects, and metabolic factors, specifically obesity and insulin resistance, in the formation of gallstones among women [2].

Histopathological examination of cholecystectomy tissues revealed notable pathological alterations in asymptomatic patients. Approximately 32% of patients had pyloric metaplasia, while 36.3% had chronic inflammatory infiltrates. These results demonstrate that asymptomatic gallstones are not entirely harmless. Gallbladder carcinoma is known to be preceded by chronic inflammation and epithelial metaplasia, especially in areas like North India that are disproportionately affected by this cancer [17]. Significantly, histological evidence of high-grade dysplastic alterations or cancer was found in 3% of instances, which included two cases of biliary intraepithelial neoplasia and one case of T2 adenocarcinoma. Consistent with previous studies, these findings highlight the significant role of gallstones in gallbladder carcinogenesis, with incidental malignancy rates of 0.3-2.9% observed in North Indian cohorts [1]. Studies have also shown that larger and more persistent stones raise the chance of developing cancer.

Indications of chronic disease were common, characterized by numerous calculi in 65.6% of cases and gallbladder enlargement in 94% of cases. Furthermore, 10% of patients had a frozen Calot's triangle, which made dissection more difficult, and 31.3% had adhesions. These anatomical difficulties support the claim that delaying surgery increases the risk and complexity of the procedure [18,19].

According to the statistical analysis, there was a significant correlation (p = 0.033) between the indication for ultrasound and histological abnormalities, but not between gender or comorbidities. This implies that dyspepsia and other moderate symptoms (e.g., nausea, urinary issues) could be early indicators of underlying pathology; however, patients remained asymptomatic for biliary colic. These findings highlight the diagnostic utility of incidental imaging in identifying gallbladder disease that is quiet but histologically aggressive.

The logistic regression revealed that age was a significant predictor of severe histopathological changes, including metaplasia or neoplasia (p = 0.014). While no specific age cut-off was identified, the risk for gallbladder cancer increased progressively with age >45 years. These findings align with global data indicating that the risk of gallbladder cancer rises with age, especially in high-prevalence regions [18].

Follow-up loss was a significant issue, with 32.3% of patients, particularly those in underserved or remote areas, failing to complete the one-month follow-up [1]. Follow-up was intended to monitor for post-cholecystectomy syndrome or recurrences, although not central to the primary histopathological outcome. This underlines the importance of proactive treatment in endemic areas, where, most of the time, diagnosis or treatment may occur at an advanced stage of the disease, leading to a poor prognosis [20,21].

The present study concludes that the existing guidelines for the treatment of asymptomatic gallstones in endemic regions need to be revised. In combination with local epidemiological statistics and demographic risk factors, these histological changes, even in the absence of symptoms, represent a strong case for elective laparoscopic cholecystectomy in a subgroup of people. This method can improve outcomes and reduce future problems in high-risk populations, such as those in North India. However, larger multicenter studies with greater numbers of subjects are needed to strengthen the recommendations.

Limitations of the study

This study has several limitations. Primarily, it is a single-center investigation with a small sample size, which may constrain the generalizability of the findings. Future studies should increase the sample population by extending the study period or collaborating with other centers. Second, the follow-up period is short, and 32 (32.30%) patients were lost to follow-up after the first postoperative visit, preventing long-term outcomes. Third, selection bias may exist as patients who opted for elective surgery were included, while those who refused were not followed. Fourth, the study lacks a control group of conservative management asymptomatic patients, so the natural history of the disease, incidence of malignant transformation, and cost-effective analysis of cholecystectomy could not be directly compared. Larger, multicentric, long-term prospective studies are needed to strengthen the findings.

## Conclusions

This prospective observational study provides clinical and histological significance of silent gallstone disease in a high-risk population in North India. A high proportion of patients were found to have chronic inflammation, epithelial metaplasia, and even premalignant or malignant changes on the histological examination. However, they never presented the classic biliary symptoms. These findings highlight the insidious progression from gallstones to gallbladder cancer. Laparoscopic cholecystectomy offers a definitive cure and is safe and effective for asymptomatic cholelithiasis; furthermore, it provides an opportunity for the earlier diagnosis of potentially fatal diseases. Age strongly predicted histological abnormalities, suggesting that demographic risk stratification could be considered when making surgical decisions.

Therefore, in areas with high gallbladder cancer incidence, particularly in women, in Northern India, selective prophylactic cholecystectomy after risk stratification may appear to be justifiable in asymptomatic patients, especially considering the low surgical morbidity of modern laparoscopic techniques. Regional guidelines may need to evolve to incorporate local epidemiological data rather than relying solely on international recommendations. Multi-center studies with longer follow-up are required to confirm these observations and guide evidence-based policy. 
